# Development of an iron-selective antioxidant probe with protective effects on neuronal function

**DOI:** 10.1371/journal.pone.0189043

**Published:** 2017-12-11

**Authors:** Olimpo García-Beltrán, Natalia P. Mena, Pabla Aguirre, Germán Barriga-González, Antonio Galdámez, Edgar Nagles, Tatiana Adasme, Cecilia Hidalgo, Marco T. Núñez

**Affiliations:** 1 Biomedical Neuroscience Institute (BNI), Faculty of Medicine, Universidad de Chile, Santiago, Chile; 2 Department of Biology, Faculty of Sciences, Universidad de Chile, Santiago, Chile; 3 Universidad Metropolitana de Ciencias de la Educación, Facultad de Ciencias Básicas, Departamento de Química, Santiago, Chile; 4 Department of Chemistry, Faculty of Sciences, University of Chile, Santiago, Chile; 5 Facultad de Ciencias Naturales y Matemáticas, Universidad de Ibagué, Ibagué, Colombia; 6 Integrative Center for Applied Biology and Chemistry (CIBQA), Universidad Bernardo O’Higgins, Santiago, Chile; 7 Department of Neuroscience, CEMC and ICBM, Faculty of Medicine, Universidad de Chile, Santiago, Chile; North Carolina State University, UNITED STATES

## Abstract

Iron accumulation, oxidative stress and calcium signaling dysregulation are common pathognomonic signs of several neurodegenerative diseases, including Parkinson´s and Alzheimer’s diseases, Friedreich ataxia and Huntington’s disease. Given their therapeutic potential, the identification of multifunctional compounds that suppress these damaging features is highly desirable. Here, we report the synthesis and characterization of *N*-(1,3-dihydroxy-2-(hydroxymethyl)propan-2-yl)-2-(7-hydroxy-2-oxo-2H-chromen-4-yl)acetamide, named CT51, which exhibited potent free radical neutralizing activity both *in vitro* and in cells. CT51 bound Fe^2+^ with high selectivity and Fe^3+^ with somewhat lower affinity. Cyclic voltammetric analysis revealed irreversible binding of Fe^3+^ to CT51, an important finding since stopping Fe^2+^/Fe^3+^ cycling in cells should prevent hydroxyl radical production resulting from the Fenton-Haber-Weiss cycle. When added to human neuroblastoma cells, CT51 freely permeated the cell membrane and distributed to both mitochondria and cytoplasm. Intracellularly, CT51 bound iron reversibly and protected against lipid peroxidation. Treatment of primary hippocampal neurons with CT51 reduced the sustained calcium release induced by an agonist of ryanodine receptor-calcium channels. These protective properties of CT51 on cellular function highlight its possible therapeutic use in diseases with significant oxidative, iron and calcium dysregulation.

## Introduction

Iron is the most abundant essential trace element present in the human body. Both Fe^2+^ and Fe^3+^ play vital roles in many biological processes, making iron a key element for plant and animal metabolic processes [1–4]. Iron, which is widely distributed in nature, via its capacity to accept and donate electrons engages in one-electron exchange reactions with the potential of producing reactive oxygen species (ROS) within cells [5]. Iron plays a central role in the biosphere via its involvement in oxygen transport, electron transfer and enzymatic reactions [6]. In particular, iron is essential for hemoglobin synthesis in vertebrate red cells and thus exerts a crucial function in the storage and transport of oxygen to tissues [7]. Through the Fenton and Haber-Weiss reactions, reduced iron (Fe^2+^) reacts with H_2_O_2_ to generate Fe^3+^ plus the highly reactive hydroxyl free radical [8]. Under physiological conditions the intracellular medium provides ample reducing power in the forms of ascorbate and reduced glutathione [9, 10], which reduce Fe^3+^ to the Fe^2+^ state. These events, all of which exhibit negative ΔG values, generate redox cycling reactions in cells that result in the production of hydroxyl radical and the consumption of reduced glutathione [8].

The brain, an organ with significant energy demands, exhibits higher iron requirements than other organs in the body [11]. Iron is an essential element for neuronal function [12]. Iron deficiency affects the activity of enzymes involved in neurotransmitter synthesis that possess iron as a prosthetic group, such as monoamine oxidases A and B, tryptophan hydroxylase and tyrosine hydroxylase [12–14]. In humans and rodents, early postnatal iron deficiency results in learning and memory impairments, which persist following iron repletion [15, 16]. Conversely, brain iron content gradually increases during normal aging and in neurodegenerative conditions such as Alzheimer’s disease (AD) and Parkinson’s disease (PD) [17]. The resulting increase in brain iron content is likely to cause increased ROS generation that can lead to irreversible damage of lipids, proteins, and DNA [8, 18]. It follows, therefore, that optimal iron levels are required for normal brain function since iron deficiency and iron excess cause neuronal dysfunction or death.

At the cellular level, iron accumulation enhances ROS production in neuroblastoma cells, which activates redox-sensitive signaling pathways [19]. Several studies in a variety of cells types indicate that ROS affect the function of proteins engaged in calcium signaling, including the highly redox-sensitive ryanodine receptor (RyR) channels that mediate calcium release from the endoplasmic reticulum [20]. Accordingly, increases in neuronal iron content enhance calcium signal generation, which if uncontrolled leads to neuronal cell death [12]. Previous studies have indicated that iron acquisition by primary hippocampal neurons generates calcium signals mediated by oxidative stimulation of RyR calcium release channels; the ensuing iron-induced calcium signals stimulate phosphorylation and nuclear translocation of extracellular signal-regulated kinases 1/2 (ERK1/2) [21], and promote significant neuronal mitochondrial fragmentation [22]. In agreement with these results, iron overload promotes mitochondrial fragmentation in mouse HT-22 hippocampal neurons via calcineurin-sensitive signals [23] and increases intracellular free calcium concentration, which leads to calcineurin activation via calcium-dependent pathways engaging calmodulin and calpain [24]. Hence, non-physiological iron-induced calcium signals, which through calcineurin activation promote mitochondrial fragmentation, are a likely factor in iron-induced neurotoxicity.

Due to the central role of iron in biological processes, the generation of fluorescent probes for iron determination has gained increased importance. Methodologies for the detection of iron include physical, chemical and biological techniques such as atomic absorption spectroscopy, colorimetry, spectrophotometry, voltammetry, and flow injection [25–30]. Fluorophores with different chemical structures, including dansyl-based probes, rhodamine-based probes, benzimidazole-based probes, imidazole-based probes, naphthyl-based probes, anthracene-appended amino acid probes, coumarin-based probes, and *N*-azacrown carbazole probes, all have been used for the detection of iron [31].

As detailed above, iron accumulation occurs frequently in neurodegenerative diseases such as AD and PD. Of note, iron chelation reduces the progression of neurological symptoms associated with these pathological conditions [17, 32]. However, multifunctional iron-chelating compounds with preference for a specific organelle are scarce since the vast majority partitions to the cytoplasm. Accordingly, a key objective of our present work was to develop a new multifunctional compound with protective properties against iron-induced oxidative cellular damage. We placed particular emphasis on generating an agent that afforded protection against mitochondrial dysfunction, oxidative damage and sustained calcium signal generation, three conditions that are deleterious to cell function.

We report here the synthesis, chemical and biological characterization of a new polyhydroxyl coumarin, *N*-(1,3-dihydroxy-2-(hydroxymethyl)propan-2-yl)-2-(7-hydroxy-2-oxo-2H-chromen-4-yl)acetamide (CT51), as a highly selective Fe^2+^ chelator and a potent antioxidant agent that preferentially partitions to mitochondria.

## Results and discussion

### I. Synthesis and properties of CT51

**Fig 1:**
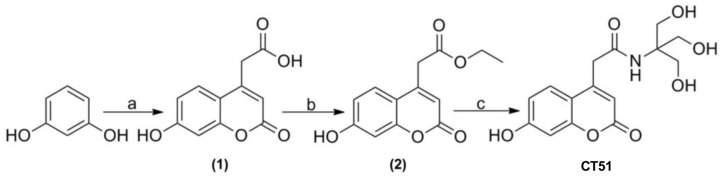
Synthetic route to produce CT51. Reagents and conditions: a) Citric acid, H_2_SO_4_, room temperature for 48 h; b) H_2_SO_4_, ethanol, reflux 6 h; c) 2-amine-2-hydroxymethyl-propane-1,3-diol, ethanol, reflux 48 h.

As illustrated in Fig 1, CT51 was synthesized from resorcinol, condensing it with citric acid (Pechmann) to obtain 2-(7-hydroxy-2-oxo-2H-chromen-4-yl) acetic acid (compound 1). To synthesize CT51, compound 1 was esterified under Fischer-Speier conditions, giving ethyl 2-(7-hydroxy-2-oxo-2H-chromen-4-yl)acetate (compound 2), which was reacted with 2-amino-2-(hydroxymethyl)propane-1,3-diol (TRIS). The resulting CT51 product was characterized by ^1^H-NMR spectroscopy (S1A Fig), ^13^C-NMR spectroscopy (S1B Fig), electrospray ionization mass spectrum (S1C Fig) and X-ray crystallography (S1D Fig and S1 Table). The presence of only 12 carbons instead of 13 atoms in the NMR spectrum stems from the fact that the signal originated by the missing carbon is hidden by the DMSO signal. This carbon atom was assigned to the methylene group, which acts as a bridge between the coumarin ring and the amide group.

The CT51 molecule is water-soluble, most probably due to the three hydroxyl groups originating from the TRIS moiety, which also comprises a putative iron coordination site. A fourth hydroxyl group is present in the phenol moiety attached to C7 in the benzopyrone ring, which acts as a strong electron donor [33, 34], leading to polarity-dependent fluorescence intensity that increases with increasing polarity of the medium with little spectral shift [35]. The absorption spectrum of CT51 displayed a maximum at 330 nm and a shoulder near 370 nm (S2A Fig). This result agrees with other absorption spectroscopic studies of 7-hydroxycoumarins, which exhibit absorption maxima in the range 320–400 nm, depending on the nature of the substituent groups at C3 or C4. Regularly, these compounds have been reported to have band groups of UV absorptions at 270–350 nm due to π–π* transitions [36]. At 330 nm, CT51 displayed a molar extinction coefficient (ε) of 17,460 M^-1^cm^-1^. Fluorescence studies revealed that CT51 had an emission band with a maximum at 460 nm (S2B Fig). The emission quantum yield (Φ) of CT51 was Φ = 0.34, determined by using quinine sulfate as standard; the lifetime (τ) was 5.95 ns, and the Stokes shift was 130 nm.

**Fig 2:**
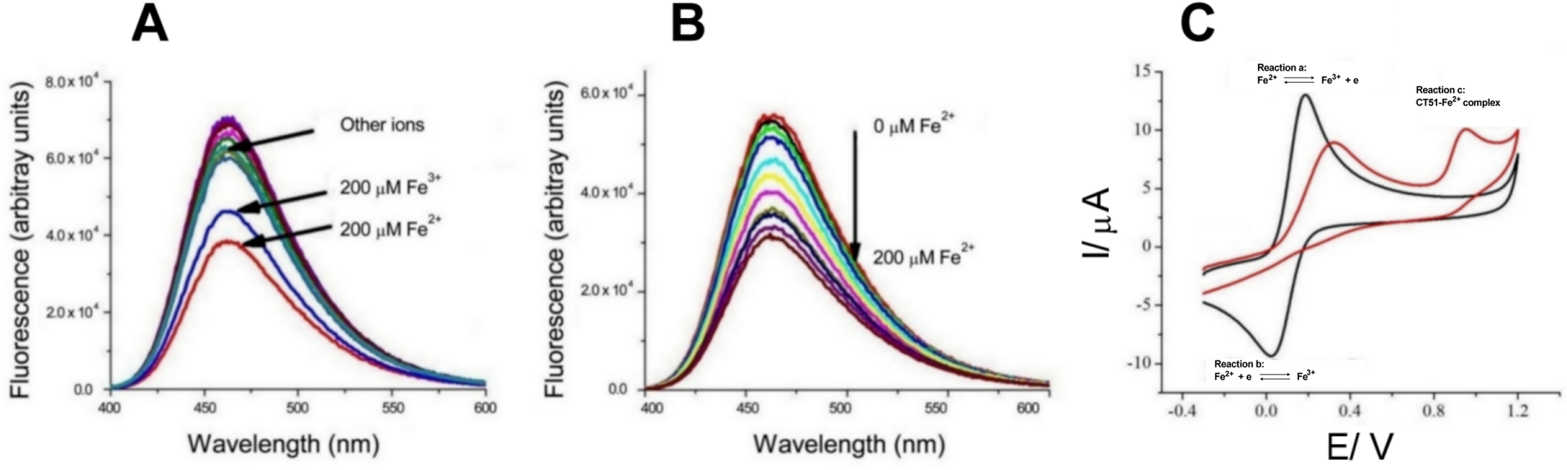
Cation selectivity of CT51. (**A**) Fluorescence emission spectra of CT51 (20 μM) recorded before and after addition of several different chloride salts of Hg^2+^, Fe^3+^, Fe^2+^, Co^2+^, Cu^2+^, Ca^2+^, Zn^2+^, Mn^2+^, Mg^2+^, Ni^2+^, Pb^2+^ or Cd^2+^; all salt solutions (200 μM) were prepared in 20 mM HEPES buffer, pH 7.4. (**B**) Emission spectra of CT51 (20 μM) were recorded (λ_ex_ = 330 nm) upon addition of increasing concentrations (up to 200 μM) of Fe(NH_4_)_2_(SO_4)2_·6H_2_O prepared in 20 mM HEPES buffer, pH 7.4. (**C**) Cyclic voltammetric analysis. The voltammograms represent the behavior of solutions containing 0.58 mM Fe^2+^ without CT51 (black line), or in the presence of 0.96 mM CT51 (red line). The voltammograms were performed at a scan rate of 100 mV s^-1^, using a potentiostat DropSens μStat 400.

The cation selectivity of CT51 was detected by the quenching of fluorescence caused by the following cations: Hg^2+^, Fe^3+^, Fe^2+^, Co^2+^, Cu^2+^, Ca^2+^, Zn^2+^, Mn^2+^, Mg^2+^, Ni^2+^, Pb^2+^ or Cd^2+^. Only Fe^2+^, and to a lesser extent Fe^3+^, produced a significant quench of CT51 fluorescence (Fig 2A). Increasing the concentration of Fe^2+^ up to the 200 μM range partially reduced the fluorescence of 5 μM CT51 (Fig 2B). The selectivity of CT51 for Fe^2+^ and Fe^3+^ makes it a possible candidate for iron chelation in living cells. A similar coumarin-TRIS compound (DAT-1) was recently characterized as a Fe^3+^ chelator [37]. At difference with CT51, DAT-1 did not demonstrate selectivity for Fe^2+^. We don’t know the causes for this discrepancy, but the use of 0.2 M citrate, a mild Fe^2+^ chelator, in the solutions used to test DAT-1 [37] possibly hindered the interaction of Fe^2+^ with DAT-1.

Cyclic voltammetry analysis represents a useful tool to study redox processes. Hence, to confirm iron chelation, we performed voltammetric assays using a Screen Printed Carbon Electrode (SPCE) and a solution of 0.58 mM Fe^2+^ in the absence (black line, pH 3.0) and in the presence of 0.96 mM CT51 (red line, pH 6.8) (Fig 2C). These assays showed an oxidation current for the reaction Fe^2+^ ↔ Fe^3+^ + e- at 0.18 V (reaction a), and a cathodic current for the reaction Fe^3+^ + e- ↔ Fe^2+^ at 0.034 V (reaction b), with ΔV = 0.146 V, indicating a quasi-reversible process. When an optimal concentration of CT51 was added to the Fe^2+^-containing solution, a decrease in the Fe^2+^ anodic current was noted. Also, a change in the potential at 0.94 V emerged (reaction c), which may be due to CT51 oxidation in the complex with Fe^3+^. Importantly, we did not observe a cathodic signal for the reaction Fe^3+^ + e- ↔ Fe^2+^. Probably, Fe^3+^ generated from the oxidation of Fe^2+^ forms a stable complex with CT51 and therefore it is not reduced back to Fe^2+^, so that in the reaction Fe^2+^ → Fe^3+^ + e-, Fe^3+^ becomes irreversibly oxidized. This is a relevant finding, since stopping Fe^2+^/Fe^3+^ redox cycling in cells should prevent the hydroxyl radical production resulting from the Fenton reaction [8]. Notwithstanding, undetermined cellular reactions could decompose the CT51-Fe^3+^ complex making Fe^3+^ available again.

The Stern-Volmer analysis yielded a linear representation (S3A Fig); based on this result we suggest that the metal-ligand (Fe^2+^-CT51) bond has 1:1 stoichiometry and that the quenching is 100% static. This latter assumption was verified by determining the τ/2 value in the presence and absence of the metal ion, as well as by determining the rate constant (kq) that displayed a value (in s^-1^) of 5.82 x 10^11^ ± 0.4 x 10^11^; kq values in this range are representative of the static quenching typical of chelators. The association constant of CT51-Fe^2+^ was determined by the Benesi–Hildebrand equation, which allows for the determination of the apparent association constant (Ka) value from the slope and intercept of the plot (S3B Fig). The Ka value for Fe^2+^ binding to CT51 was 6.63 x 10^−4^ M^-1^. The constant slope of the Benesi–Hildebrand plot supports the above proposal of 1:1 stoichiometry for the complex CT51-Fe^+2^. The detection limit of this Fe^2+^ fluorescent sensor was 1.1 x 10^−7^ M, which allows for the detection of the labile iron pool in the cell cytoplasm that presents values in the low 10^−6^ M range [38, 39].

**Fig 3:**
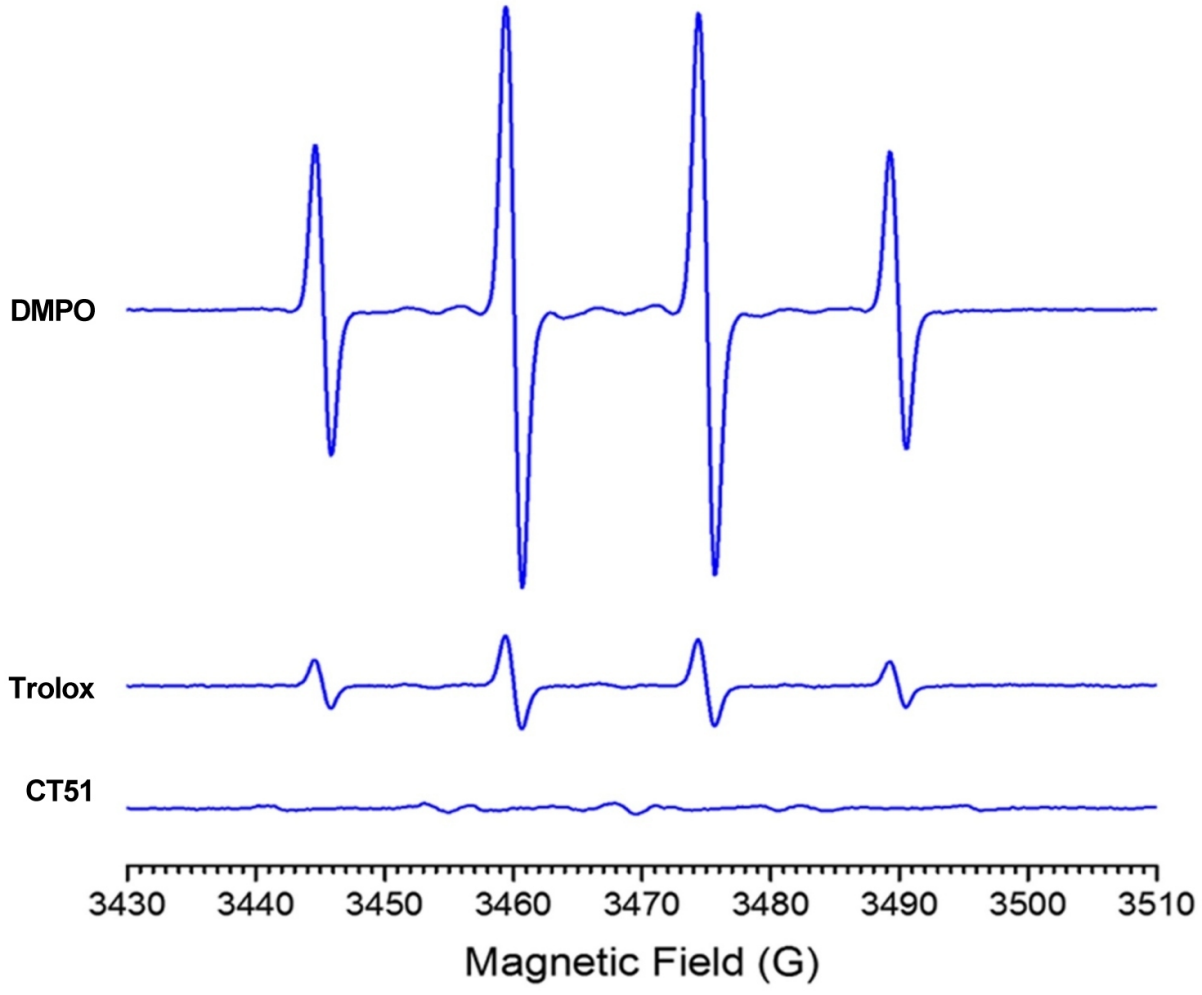
CT51 is a free radical quencher. EPR spectra of the spin adduct DMPO recorded in the absence or presence of CT51 (3 mM) or of the antioxidant agent Trolox (3 mM).

Since antioxidant properties are a desired trait of neuroprotective agents, we evaluated next through chemical and biological assays the putative antioxidant capacity of CT51. To explore the antioxidant capacity of CT51, we determined the EPR spectra of 5,5-dimethyl-1-pyrroline-*N*-oxide (DMPO) in the absence or presence of CT51. DMPO is a spin trap widely used to study the levels of oxygen-based free radicals. Superoxide, hydroxyl and thiyl radicals form adducts with DMPO that can be detected with EPR spectroscopy. Hydroxyl radicals were generated by the Fenton reaction in the presence of DMPO, or DMPO plus Trolox or CT51 as hydroxyl radical competitors. As positive control for free radical quenching we used the antioxidant agent 6-hydroxy-2,5,7,8-tetramethylchroman-2-carboxylic acid (Trolox), a gold standard for oxygen radical absorbance capacity [40]. The resulting EPR spectra indicated that CT51 possesses considerable capacity for free radical quenching (Fig 3). The values of the percentage reduction of the signal, calculated using the double integral of the EPR spectra, were DMPO: 0.0, Trolox: 84.8 and CT51: 99.8. In addition, voltammogram studies of CT51, determined at pH 6.8 or pH 3.0 (S4 Fig) showed that at pH 3.0, the oxidation current increased and the potential shifted from 0.71 V, indicating involvement of the phenolic proton of CT51 during the oxidation process. This proton has the capacity to neutralize ROS belonging to the free radical category [41].

### II. CT51 does not impair cell viability and protects cells from oxidative stress

**Fig 4:**
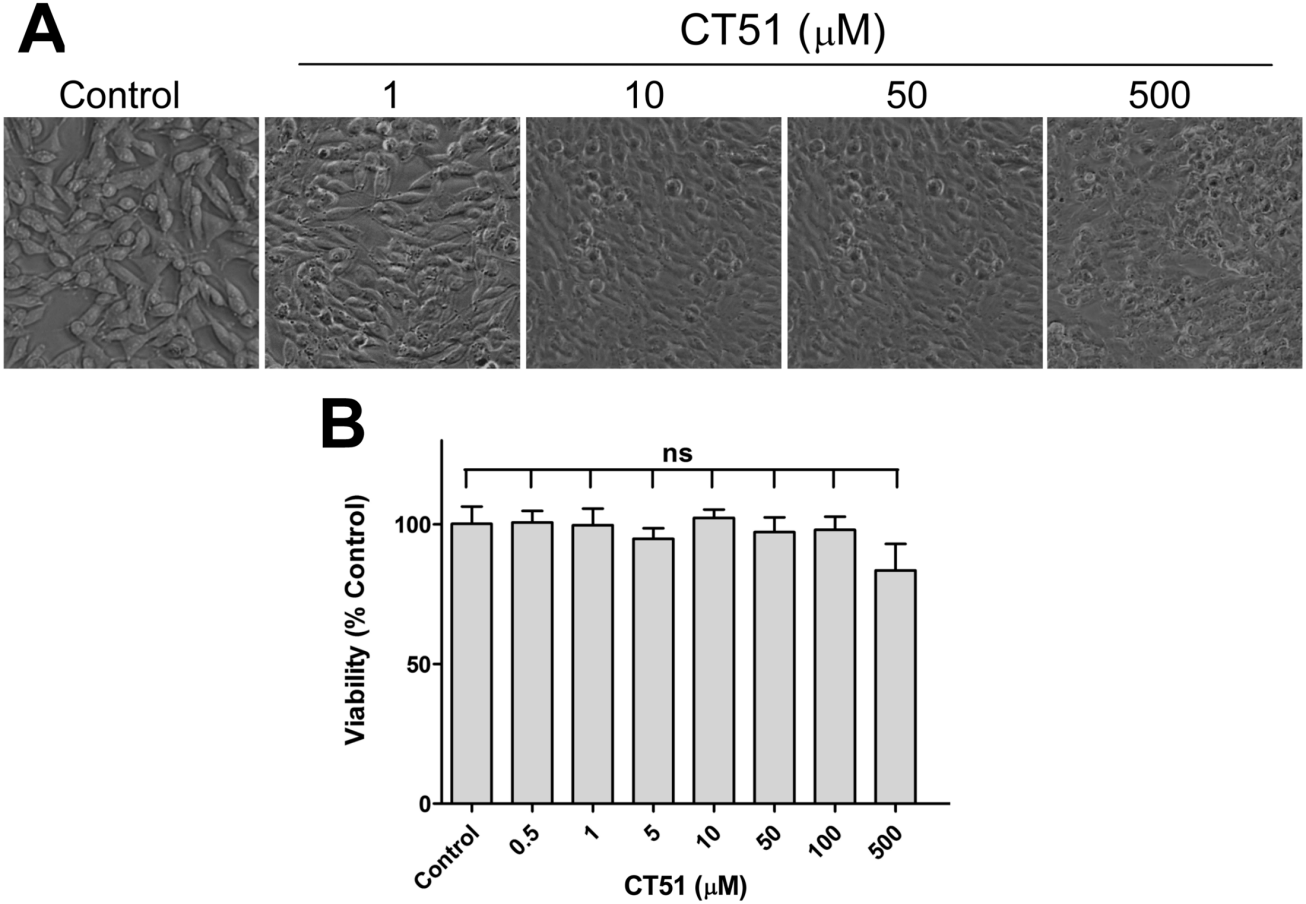
CT51 does not affect cell viability. Cell viability was determined in SH-SY5Y cells incubated for 48 h with varying concentrations of CT51. (**A**) Representative images of the cell culture after 48 h of treatment with CT51. (**B**) Cell viability determined by the MTT assay. Values represent Mean ± SEM from three independent experiments. Significance of differences was evaluated by one-way ANOVA followed by Tukey’s post-hoc test; ns, not significant.

SH-SY5Y cells derived from a human neuroblastoma are used often as a robust *in vitro* model for the evaluation of neurotoxicity induced by candidate drugs [42, 43]. To evaluate possible neurotoxic effects of CT51, we incubated cultured SH-SY5Y human neuroblastoma cells with CT51 and tested cell viability as a function of CT51 concentration. These assays revealed that CT51 was not toxic in the 0.1–10 μM range although a change to a more rounded shape was apparent (Fig 4A and 4B).

**Fig 5:**
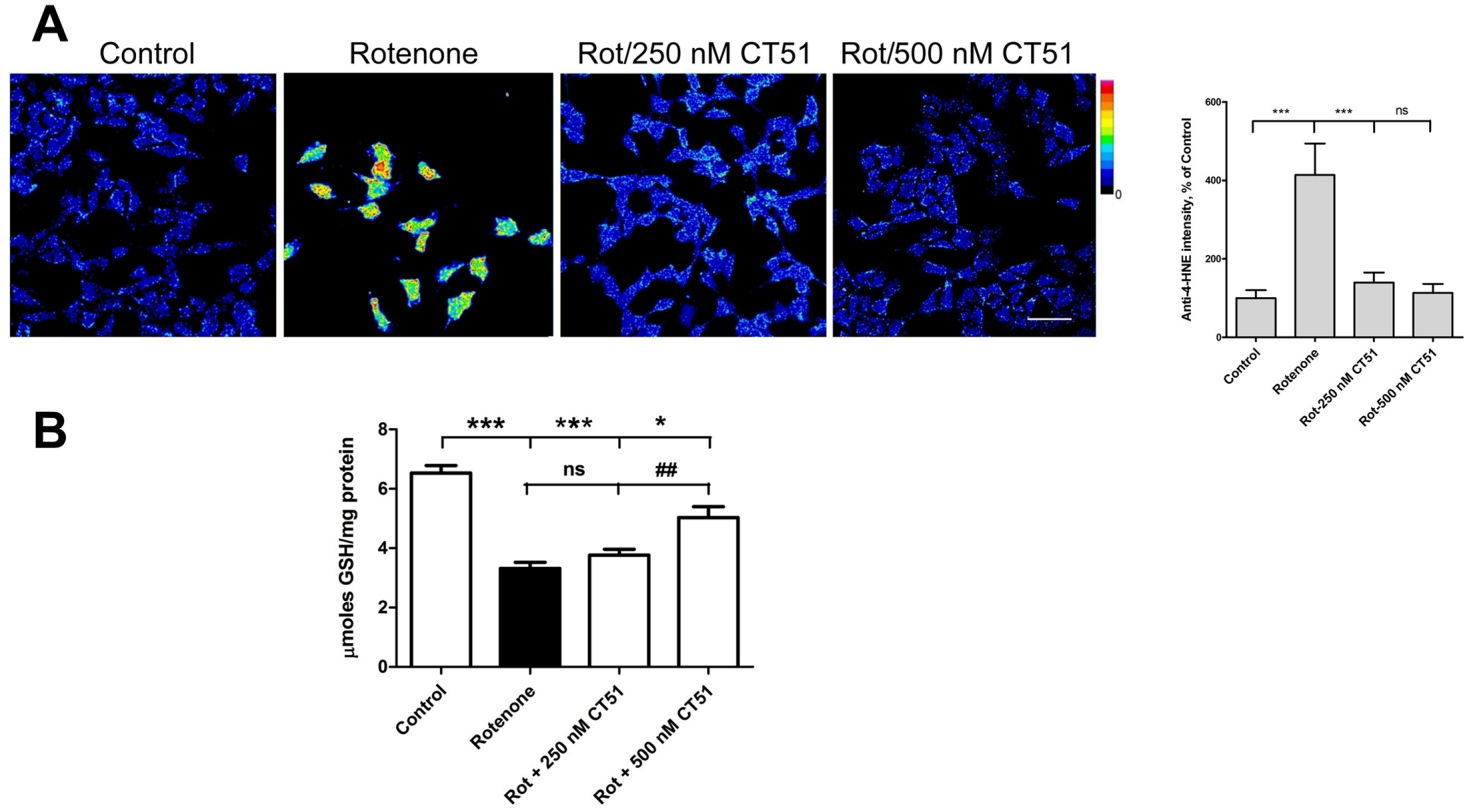
CT51 acts as a cellular antioxidant agent. (**A**) Immunofluorescence assays were performed to determine 4-HNE-protein adducts under basal culture conditions (Control) and in cultures treated for 24 h with the oxidative stress inductor rotenone (3 μM), in the absence or presence of 250 or 500 nM CT51. The figure shows images from a representative experiment out of 3 independent determinations. Rotenone treatment increased cell immunostaining of 4-HNE-protein adducts (center-left hand panel); this increase was prevented by the presence of 250 or 500 nM CT51 (center-right and right hand panels). To appreciate these differences better, the immunostaining intensity was transformed into a thermal scale with the ImageJ program (right-hand color bar), in which fluorescence intensity increases from blue to red. The right-hand graph shows the quantification of fluorescence intensity determined in 70–90 cells per experimental condition. Values represent Mean ± SD; n = 3. Significance of differences was evaluated by one-way ANOVA followed by Tukey post-hoc test. ***: p < 0.001 compared to the rotenone condition; ns: not significant. Scale bar: 30 μm. (**B**) GSH levels determined in cells treated for 24 h with the oxidative stress inductor rotenone (3 μM), in the absence or presence of 250 or 500 nM CT51. Values represent Mean ± SE; n = 3. Significance of differences was evaluated by one-way ANOVA followed by Tukey post-hoc test; ***: p < 0.001, *: p < 0.05 compared to the control condition. ^##^: p < 0.01 compared to the rotenone condition; ns: not significant.

We tested next the antioxidant capacity of CT51 in SH-SY5Y human neuroblastoma cells. The potential to modify lipid peroxidation was detected by the formation of 4-hydroxynonenal (HNE)-protein adducts (Fig 5A). HNE is a highly reactive aldehyde resulting from lipid peroxidation that quickly forms HNE-protein adducts [44]. Oxidative stress was induced by incubation of neuroblastoma cells with the mitochondrial complex I inhibitor rotenone [45]. Treatment with rotenone significantly increased the formation of HNE-protein adducts compared with the control situation; this increase was completely abolished by 500 nM CT51, while 250 nM CT51 afforded partial protection (Fig 5A). Complex I inhibition generates ROS that result in oxidative stress and decreased reduced glutathione (GSH) levels [46, 47]. Under this consideration, we tested the putative capacity of CT51 to protect against GSH depletion induced by complex I inhibition. Incubation of SH-SY5Y cells for 24-h with rotenone to inhibit complex I caused a marked decrease in GSH content (Fig 5B). Co-treatment with 500 nM CT51 largely prevented this decrease, while co-treatment with 250 nM CT51 increased GSH levels but this increase was not statistically significant (Fig 5B). We interpret these combined results as a clear indication that CT51 exerts protective effects against the oxidative damage induced by mitochondrial complex I inhibition. This CT51 property is common with that of other substituted coumarins, which have the capacity to inhibit lipid peroxidation and to scavenge hydroxyl radicals [48, 49].

The question arose as to whether redox-active copper could also mediate rotenone-induced oxidative damage. We tested this possibility by studying the effect of the high affinity, membrane-permeant copper chelator bathocuproine [50]. As opposed to CT51, bathocuproine (250–500 nM range) did not prevent the effect of rotenone on the formation of HNE-protein adducts (S5 Fig). This result is consistent with previous reports showing that under physiological conditions cellular copper chaperones keep free Cu ions in the zeptomolar range [51, 52]. This feature of copper cellular homeostasis contrasts with iron, which is present in the cellular reactive pool in the micromolar range [38, 39].

Overall, the above results indicate that CT51 has strong antioxidant capacity by which it inhibits *in vitro* the formation of DMPO-hydroxyl radical spin adducts while it protects cells from lipid peroxidation and the associated decrease in GSH content. Probably, CT51 protects cells from oxidative damage by two independent mechanisms, namely 1) by sequestering redox-active iron and 2) by neutralization of free radicals, although in turn, iron chelation should have incidence over hydroxyl radical production.

### III. Cellular distribution and iron-sensing properties of CT51

**Fig 6:**
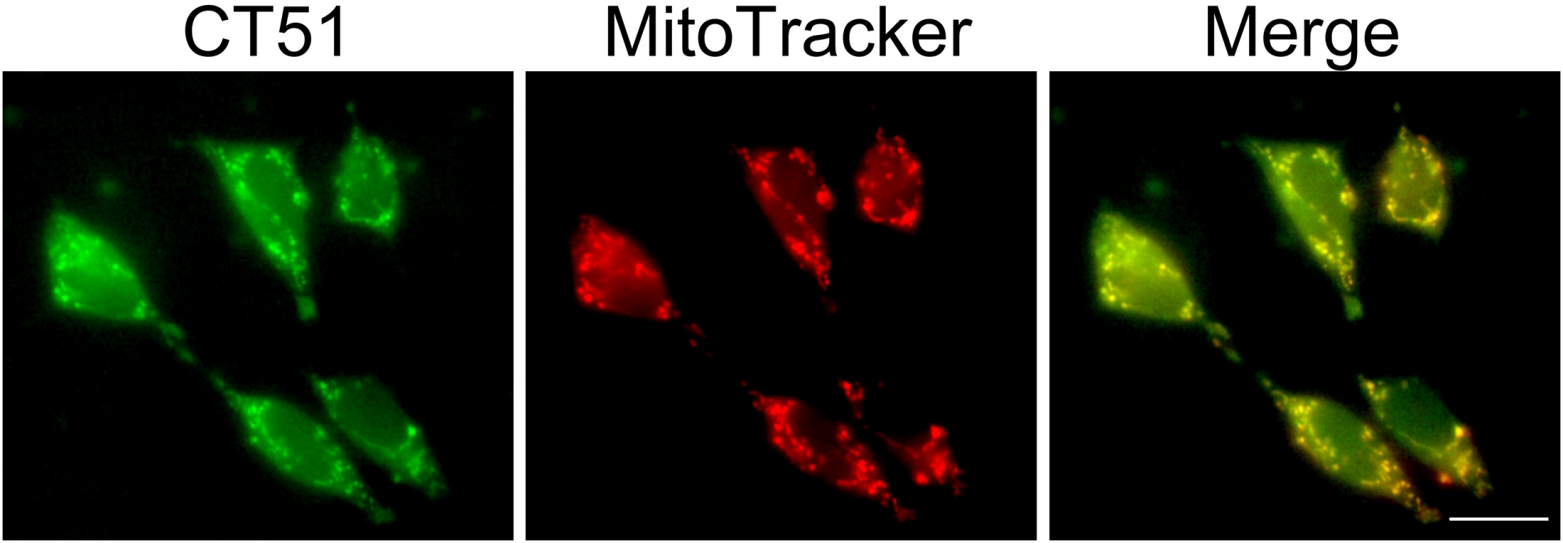
CT51 fluorescence in neuroblastoma cells. SH-SY5Y cells were loaded for 20 min with the mitochondrial tracer Mito-Tracker (A, red) plus 5 μM CT51 (B) (green). The merge image (C) shows CT51 distribution in both mitochondria (yellow) and cytoplasm (green).

Multifunctional iron chelator/antioxidant compounds with preference for a specific cell organelle are scarce, since the vast majority of membrane-permeant chelating reagents partitions to the cytoplasm. Under this consideration, we determined the cellular distribution of CT51 in SH-SY5Y human neuroblastoma cells. Using epifluorescence microscopy, we first determined its intracellular distribution taking advantage of its intrinsic fluorescence. Initial observations showed a punctate pattern reminiscent of mitochondria, so its putative mitochondrial distribution was evaluated next (Fig 6). As shown in a representative experiment, mitochondria labeled with Mito-Tracker distributed in a perinuclear pattern. The fluorescence due to CT51 (green) was also present into perinuclear organelles that co-localized with Mito-Tracker (Fig 6, Merge, yellow). CT51 also distributed in the cytoplasm, an indication that in cells CT51 exhibits both mitochondrial and cytoplasmic distribution. Arguably, distribution of CT51 to the mitochondrion should decrease the risk of oxidative damage in this organelle, considering that mitochondria contain redox-active iron and continuously generate ROS, a propitious environment for hydroxyl radical generation and the ensuing hydroxyl radical-mediated damage [53].

**Fig 7:**
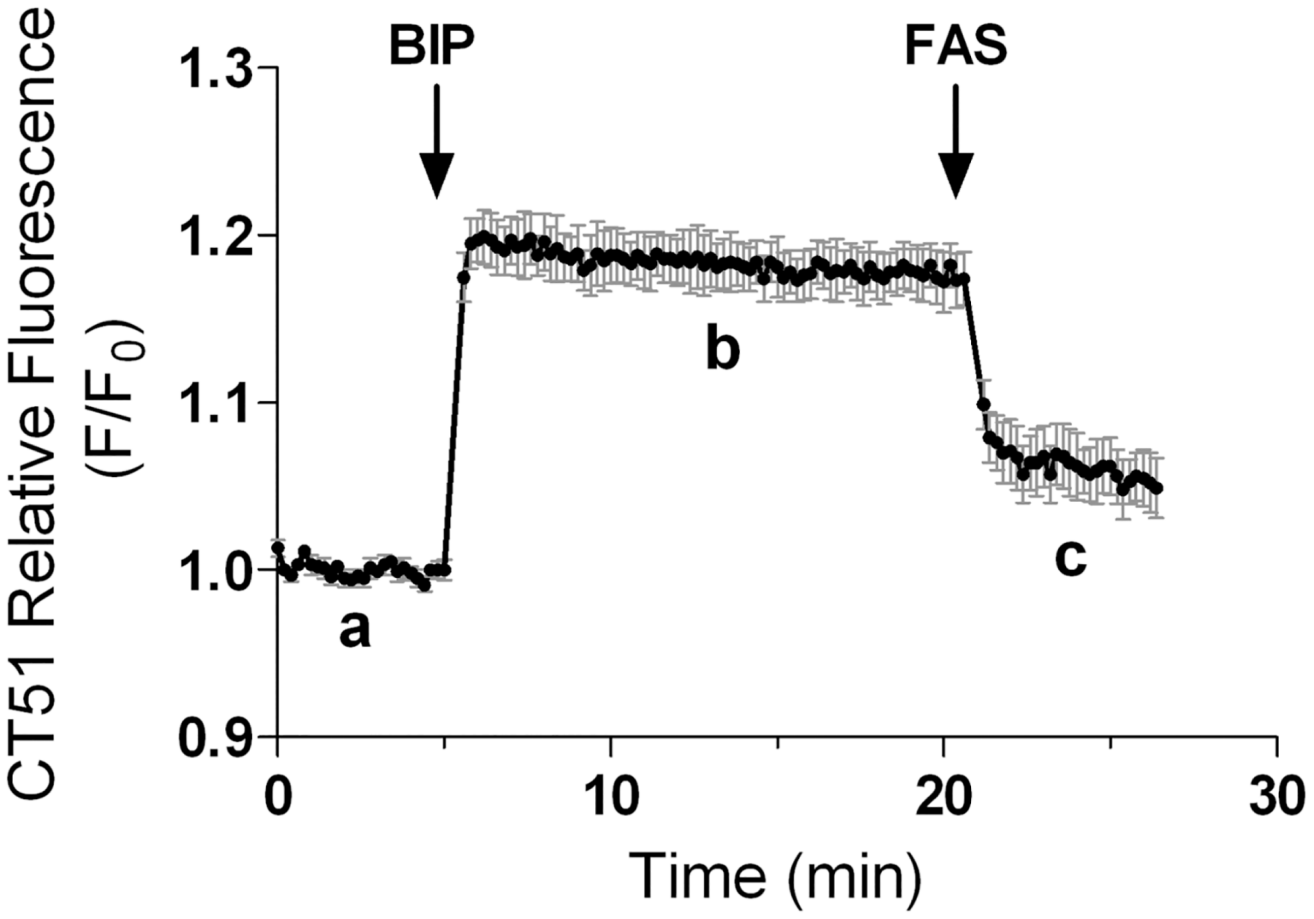
CT51 is a reversible intracellular iron sensor. The graph shows the fluorescence recorded in SH-SY5Y cells incubated with CT51 (20 min, 10 μM) and detected in a fluorescence plate reader (λ Ex. 340 nm; λ Em. 460 nm). (**a**) Basal CT51 fluorescence in cells cultured for 24 h with 20 μM Fe. (**b**) CT51 fluorescence recorded after addition of the Fe^2+^ chelator bipy (20 μM). (**c**) CT51 fluorescence recorded after addition of 80 μM FAS. Values represent Mean ± SEM; n = 3.

To determine the iron sensing properties of CT51 we measured its intracellular fluorescence upon modification of the labile iron pool in SH-SY5Y cells (Fig 7). Basal CT51 fluorescence (trace a) increased after addition of the Fe^2+^ chelator 2,2’-bipyridyl (bipy) (trace b), an indication of CT51 de-quenching after losing iron to bipy. Next we tested reversibility, another important feature of a good chemical probe, by the subsequent addition of ferrous ammonium sulfate (FAS) (trace c). Addition of FAS decreased CT51 fluorescence, probably through the re-formation of the CT51-Fe^2+^ complex.

### IV. Effects of CT51 on calcium signals elicited by RyR channel activation

**Fig 8:**
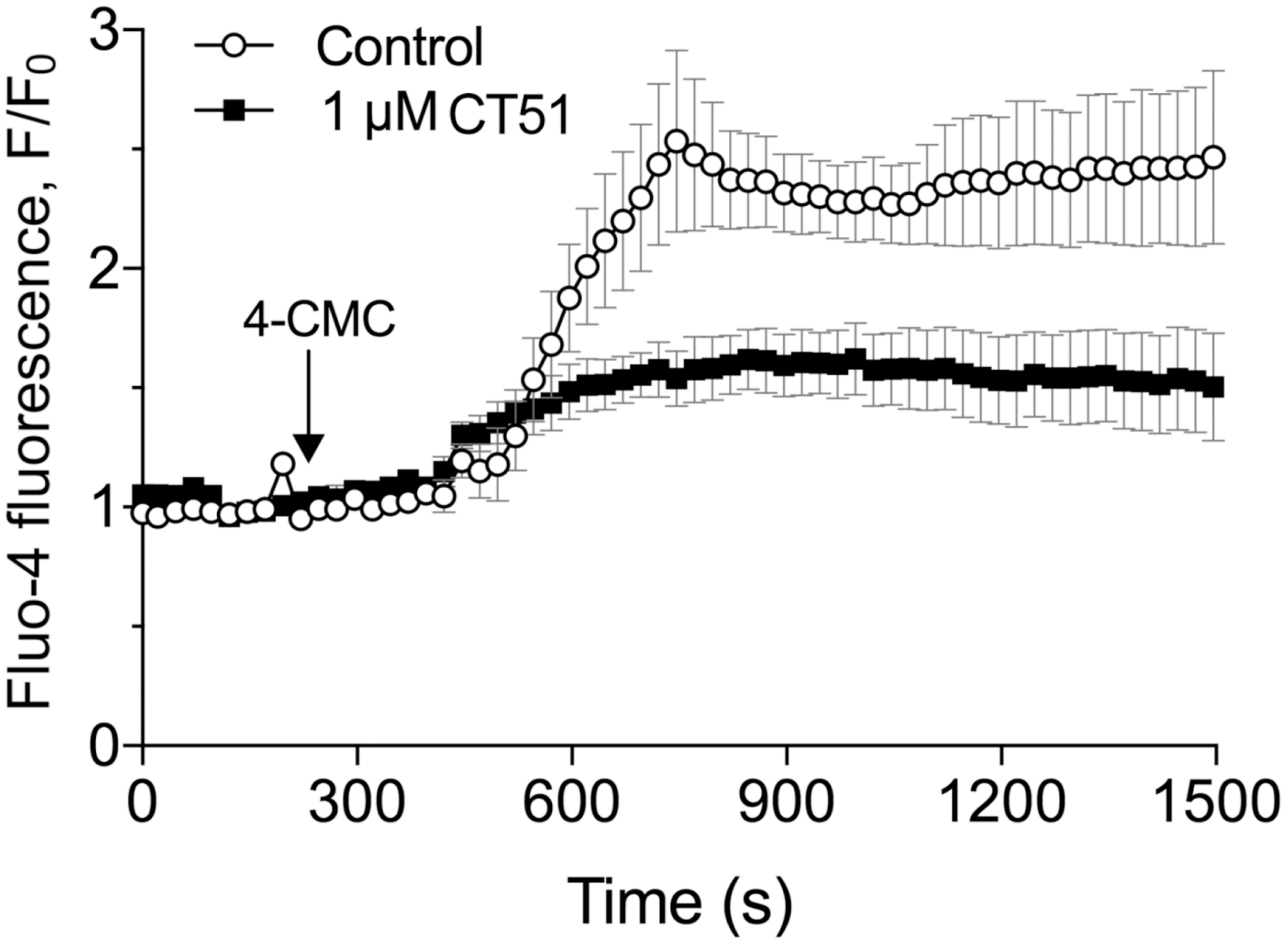
CT51 inhibits the sustained Ca^2+^ increase induced by the RyR-agonist 4-CMC. Ca^2+^ signals elicited by the RyR agonist 4-CMC in rat primary hippocampal neurons incubated for 24 h in the absence (open symbols) or the presence of 1 μM CT51 (solid symbols) were determined as a function of time. The graph shows the fluorescence values expressed as F/F_0_, where F_0_ represents the basal fluorescence recorded before 4-CMC addition. Values represent Mean ± SE; n = 3.

We tested next the effect of CT51 on another cellular component sensitive to oxidative stress, namely RyR calcium release channels; these channels reside in the endoplasmic reticulum and their activity is highly redox sensitive [54]. Many reports indicate that sustained increments of intracellular calcium levels in several neurodegenerative disorders prompt cell death [55–57]. Considering that the antioxidant agent N-acetylcysteine (NAC) prevents the high and sustained intracellular calcium increases generated by the RyR agonist 4-chloro-m-cresol (4-CMC) [58], we tested whether CT51, through its antioxidant properties, had similar effects as NAC. Addition of 4-CMC induced a persistent 2.4-fold increase of the calcium signal, recorded in the soma of rat primary hippocampal neurons (Fig 8). Pre-incubation for 24 h with 1 μM CT51 markedly decreased the calcium signal evoked by 4-CMC to 1.6-fold the initial value (57% inhibition), whereas pre-incubation with 10 μM CT51 abolished the calcium increase elicited by 4-CMC (data not shown). These results highlight CT51 as a suitable agent to reduce the neuronal calcium dysregulation caused by oxidative conditions.

## Conclusions

In summary, we report here the synthesis, chemical and biological characterization of the polyhydroxycoumarin CT51. In vitro, CT51 exhibited 1) selective Fe^2+^ and Fe^3+^ binding and 2) significant capacity to quench free radicals. In neuroblastoma cells, CT51 1) bound iron reversibly, 2) distributed to both mitochondria and cytoplasm and 3) protected against oxidative damage, while in primary hippocampal neurons, CT51 reduced the long-lasting increase in intracellular calcium levels produced by an agonist of redox-sensitive RyR channels. Based on these combined results, we propose that CT51 has the potential to function both as selective intracellular iron sensor and as protective agent against oxidative cellular damage. In particular, CT51 may protect neuronal cells *in vivo* against the coupled generation of iron-induced anomalous calcium signals, which presumably play a significant role in the progression of iron-mediated neuronal cell death and iron-related neurodegeneration. Hence, the results presented in this study may for the basis for the development of a novel iron-chelation therapy against iron-associated neurodegenerative disorders.

## Materials and methods

### Reagents

Salts (FeNH_4_(SO_4_)_2_·12 H_2_O, CaCl_2_, CuCl_2_, CoCl_2_, MgCl_2_, MnCl_2_, ZnCl_2_, PbCl_2_, CdCl_2_, HgCl_2_, solvents (analytical grade and spectroscopic grade) and other chemical reagents (analytical grade and spectroscopic grade) such as 5,5-Dimethyl-1-pyrroline-N-oxide (DMPO), 4-(2-Hydroxyethyl)piperazine-1-ethanesulfonic acid (HEPES) and (±)-6-Hydroxy-2,5,7,8-tetramethylchromane-2-carboxylic acid (Trolox) were purchased from Sigma-Aldrich; Hydrogen peroxide 30% was purchased from Merck. Ferrous ammonium sulfate hexahydrate was purchased from Mallinckrodt Baker. Stock solutions (200 μM) of the metal ions were prepared in Milli-Q water with the salts Fe(NH_4_)_2_(SO_4_)_2_·6H_2_O, FeNH_4_(SO_4_)_2_·12H_2_O, CaCl_2_, CuCl_2_, CoCl_2_, MgCl_2_, MnCl_2_, ZnCl_2_, PbCl_2_, CdCl_2_, or HgCl_2_.

### Spectroscopy

^1^H-NMR and ^13^C-NMR spectra were recorded on a Bruker multidimensional 400 MHz spectrometer, using the solvent or the TMS signal as an internal standard. All chemical shifts are reported in the standard δ notation of parts per million. EPR spectra were recorded in the X band (9.80 GHz) using a Bruker ECS 106 spectrometer with a rectangular cavity and 50 KHz field modulation. Absorption spectra were recorded at 25°C using a Perkin Elmer model Lambda 11 spectrometer. Voltammograms were obtained using a potentiostat DropSens μStat 400. To perform this analysis, 7.5 mL of ultrapure water, 0.5 mL Phosphate-buffered saline, pH 3.0 or 6.8, and 0.25 mL of 1.54 mM CT51 were added to the electrochemical cell. After a resting time of 3 s, the voltammograms were performed at a scan rate of 100 mV s^-1^.

### Synthesis of CT51

Resorcinol was condensed with citric acid (Pechmann) [59] obtaining the compound 2-(7-hydroxy-2-oxo-2*H*-chromen-4-yl)acetate (compound **1**). Subsequently, compound **1** was esterified according to Fischer-Speier [60], generating 2-(7-hydroxy-2-oxo-2*H*-chromen-4-yl)acetate (compound **2**), which was reacted with 2-amino-2-(hydroxymethyl) propane-1, 3-diol (TRIS) to obtain, by analogy to a published procedure (Jones et al, 1985), *N*-(1,3-dihydroxy-2-(hydroxymethyl)propan-2-yl)-2-(7-hydroxy-2-oxo-2H-chromen-4-yl)acetamide (CT51). The CT51 molecule, isolated by column chromatography using as mobile phase ethyl acetate:MeOH 9:1 (0.9 g, 70%), was crystallized in methanol. The melting point of solid CT51, determined on a Reichert-Jung Galen III equipped with a thermocouple, was 145–147°C. ^1^H-NMR spectroscopy of CT51 (400 MHz, DMSO-*d*_*6*_) yielded: 7.65 (s, 1H), 7.62 (d, 1H, *J* = 8.6), 6.76 (dd, 1H, *J* = 8.6, 2.0), 6.69 (d, 1H, *J* = 2,0), 6.17 (s, 1H), 4.7 (br, 3H), 3.7 (s, 2H), 3.55 (s, 6H). ^13^C-NMR spectroscopy of CT51 (100 MHz, DMSO-*d*_*6*_) yielded: δ 60.8, 63.0, 102.7, 111.8, 113.5, 127.3, 152.1, 155.5, 160.8, 162.0, and 169.3. The *m/z* observed value was 346.0897, and the calculated value for C_15_H_17_NO_7_Na was 346.0903.

### Deactivation of fluorescence

Deactivation of fluorescence was analyzed by the Stern-Volmer equation [59], [61], defined in S1 Appendix.

### Benesi-Hildebrand plot analysis

Fluorescence intensity data for the CT51-Fe^2+^ complex were plotted according to the Benesi-Hildebrand equation [62], defined in S1 Appendix.

### Calculation of fluorescence quantum yield

The fluorescence quantum yield (Φ) was determined using quinine sulfate dissolved in 0.5 M H_2_SO_4_ (Φr = 0.546) as standard [63], and was calculated using the equation defined in S1 Appendix.

### Free radical generation and EPR spectroscopy

A competition assay was performed to determine and compare the antioxidant capacities of the vitamin E analogue Trolox, a known free radical quencher [64], with those of compound CT51 synthesized in this work. For this purpose, hydroxyl radical was first generated through the Fenton reaction using an aqueous Fe^2+^ solution (1.0 mM aqueous ferrous ammonium sulfate hexahydrate), combined with an aqueous solution of hydrogen peroxide (1%) in HEPES buffer, pH 7.3. A control was performed by trapping the hydroxyl radical with DMPO (50 mM) and a total of 12 scans per spectra were acquired [65]; this control was assigned a 100% value for the hydroxyl radical signal. Next, the effects of Trolox or CT51 on the above signal were determined. EPR spectra were recorded in the X band (9.80 GHz) using a Bruker ECS 106 spectrometer with a rectangular cavity and 50 KHz field modulation. All spectra were in the same scale and the most resolved line was selected to obtain the area under the curve through the double integration of the selected signal.

### SH-SY5Y cells and primary hippocampal neuronal cultures

Human neuroblastoma SH-SY5Y cells (CRL-2266, American Type Culture Collection, Rockville, MD) were cultured at low humidity, at 37°C and 5% CO_2_, in Dulbecco’s modified essential medium (DMEM)-F12 medium supplemented with 10% fetal bovine serum, non-essential amino acids, antibiotic-antimycotic mixture, and 20 mM HEPES buffer, pH 7.2. The medium was replaced every 2 days. Cells were used at day 5 of culture. Primary rat hippocampal cultures were prepared from eighteen-day old embryos obtained from pregnant Sprague-Dawley rats as previously described [58]. Briefly, brains were removed and placed in a dish containing Hank’s-glucose solution. Hippocampi were dissected and neurons were gently mechanically dissociated in Hank’s-glucose solution, centrifuged and suspended in DMEM supplemented with 10% horse serum. Dissociated hippocampal neurons were plated on polylysine-treated glass coverslips. After 40 minutes, DMEM was replaced by Neurobasal medium supplemented with B-27. Cells were incubated for 14 days in vitro (DIV) at 37°C in a humidified 5% CO_2_ atmosphere prior to experimental manipulations. All experimental protocols used in this work complied with the “Guiding Principles for Research Involving Animals and Human Beings” of the American Physiological Society and were approved by the Bioethics Committee on Animal Research, Faculty of Medicine, Universidad de Chile.

### Mitochondrial localization of CT51

SH-SY5Y cells grown in 12-well plates for 5 days were incubated for 15 min with the mitochondrial probe Mito-Tracker Orange (0.5 μM) (Thermo-Fisher Scientific) and CT51 (25 μM). After washing the probes, the fluorescence intensity of Mito-tracker and CT51 was detected using a Zeiss Axiovert epifluorescence inverted microscope. Co-staining between CT51 and Mitotracker was determined with the ImageJ program.

### Immunodetection of HNE-protein adducts

HNE immunodetection was performed as described [66]. Briefly, SH-SY5Y cells grown in glass coverslips were treated for 24 h with the oxidative stress inductor rotenone (3 μM) in the absence or presence of 250 or 500 nM CT51, or of 250 or 500 nM of the Cu^+1^ chelator bathocuproine. Protein-HNE adducts were detected with mouse monoclonal anti-4-HNE antibody (J-2, Abcam, dilution 1:500) as a primary antibody and Alexa-488-conjugated anti-mouse as secondary antibody. Fluorescence intensity of HNE adducts was detected in a Zeiss Axiovert epifluorescence inverted microscope.

### GSH determination

Total GSH content was quantified using the enzymatic recycling method adapted to microplate readers as described [67, 68].

### Intracellular iron sensing by CT51

SH-SY5Y cells grown in 12-well plates for 5 days were incubated with CT51 (20 min, 10 μM). After washing of CT51, basal fluorescence (λ Ex. 340 nm; λ Em. 460 nm) was recorded in a BioTek Gen5 microplate fluorescence reader. To de-quench CT51 fluorescence, the Fe^2+^ chelator bipy (20 μM) that competes with CT51 for iron, was added to the cells. CT51 fluorescence was subsequently re-quenched by addition of 80 μM ferrous ammonium sulfate (FAS).

### Changes in intracellular calcium levels

Primary hippocampal neurons were pre-incubated for 12 h with 1 μM CT51 and were then loaded with the fluorescent calcium probe Fluo-4 AM [58]. Fluorescence changes as a function of time were followed in a Zeiss 710 confocal fluorescence microscope.

## Supporting information

S1 Fig Spectral properties of CT51. (**A**) ^1^H-NMR spectrum. (**B**) ^13^C-NMR spectrum. (**C**) ESI-MS analysis of CT51. (**D**) Crystal structure of CT51. Crystallographic data (excluding structure factors) for the structural analysis have been deposited in the Cambridge Crystallographic Data Centre, CCDC 897001. These data can be obtained free of charge from the Cambridge Crystallographic Data Centre; Postal Address: CCDC, 12 Union Road, Cambridge CB21EZ, UK, Phone: (44) 01223 762910, Fax: (44) 01223 336033, e-mail: deposit@ccdc.cam.ac.uk.
(PDF)

S2 Fig Absorption and emission spectra of CT51. (**A**) Absorption spectra. (**B**) Emission spectra.
(PDF)

S3 Fig Stern-Volmer and Benesi-Hildebrand analysis. (**A**) Stern-Volmer relation for CT51 in the presence of Fe^2+^. Deactivation of fluorescence was analyzed by the Stern-Volmer equation. (**B**) Benesi-Hildebrand plot for CT51 with added Fe^2+^. Fluorescence intensity data for the CT51-Fe^2+^ complex were plotted according to the Benesi-Hildebrand equation.
(PDF)

S4 Fig Voltammograms of CT51 at pH 6.8 and pH 3. Voltammograms of CT51 (0.96 mM) determined at pH 6.8 (red line) or pH 3.0 (black line). To perform the cyclic voltammetry analysis, 7.5 mL of ultrapure water, 0.5 mL Phosphate-buffered saline, pH 3.0 or 6.8, and 0.25 mL of 1.54 mM CT51 were added to the electrochemical cell. After a resting time of 3 s, the voltammograms were performed at a scan rate of 100 mV s^-1^.
(PDF)

S5 Fig Cu is not involved in rotenone-induced 4-HNE-protein adduct formation. (**A**) Immunofluorescence of 4-HNE-protein adducts formed under basal culture conditions (Control) and in cultures treated for 24 h with the oxidative stress inductor rotenone (3 μM), in the absence or presence of 250 or 500 nM of the Cu^+1^ chelator bathocuproine (BC) or CT51. The figure shows images from representative experiments out of 2 independent determinations. Rotenone treatment increased cell immunostaining of 4-HNE-protein adducts. This increase was partially prevented by 250 nM CT51 and completely prevented by 500 nM CT51 but not by 250 or 500 nM bathocuproine. To appreciate these differences better, the immunostaining intensity was transformed into a thermal scale with the ImageJ program (right-hand color bar), in which fluorescence intensity increases from blue to red to white. (**B**) Quantification of fluorescence intensity determined in 25–45 cells per experimental condition. Values represent Mean ± SEM. Significance of differences was evaluated by one-way ANOVA followed by Tukey’s post-hoc test; ns: not significant.
(PDF)

S1 Appendix Stern-Volmer equation (A), Benesi-Hildebrand equation (B) and equation used to calculate the fluorescence quantum yield (C). (PDF)

S1 Table Crystal data and details of CT51 structure determination. (PDF)
