# Supplementary material for: Development of an iron-selective antioxidant probe with protective effects on neuronal function
Source: PLoS One. 2017 Dec 11;12(12):e0189043. doi: 10.1371/journal.pone.0189043 (PMC5724820; doi:10.1371/journal.pone.0189043)
Supplement: S1 Fig — (A) 1H-NMR spectrum. (B) 13C-NMR spectrum. (C) ESI-MS analysis of CT51. (D) Crystal structure of CT51. Crystallographic data (excluding structure factors) for the structural analysis have been deposited in the Cambridge Crystallographic Data Centre, CCDC 897001. These data can be obtained free of charge from the Cambridge Crystallographic Data Centre; Postal Address: CCDC, 12 Union Road, Cambridge CB21EZ, UK, Phone: (44) 01223 762910, Fax: (44) 01223 336033, e-mail: deposit@ccdc.cam.ac.uk. (PDF) [file pone.0189043.s001.pdf]

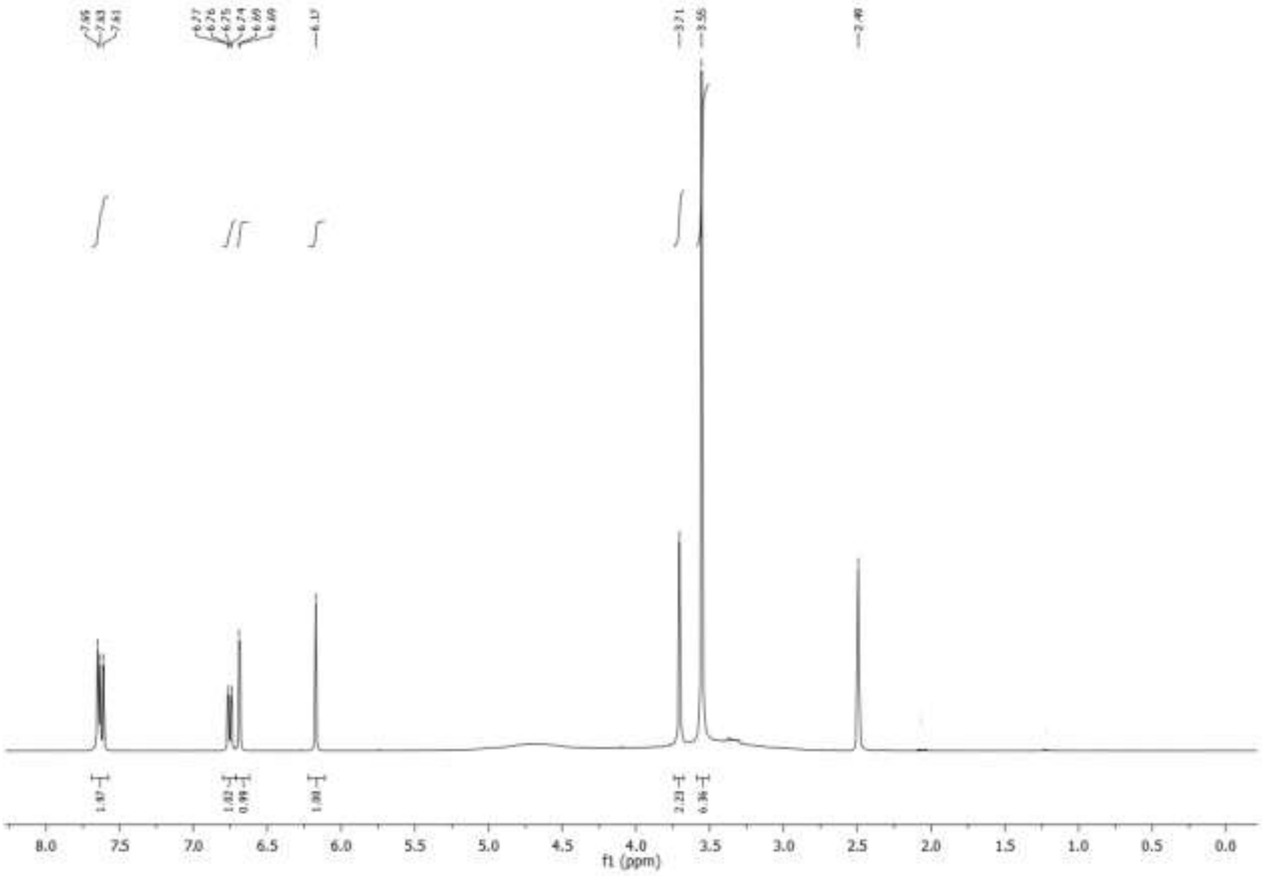

S1A Fig. Spectral properties of CT51. ^{1}H-NMR spectrum.

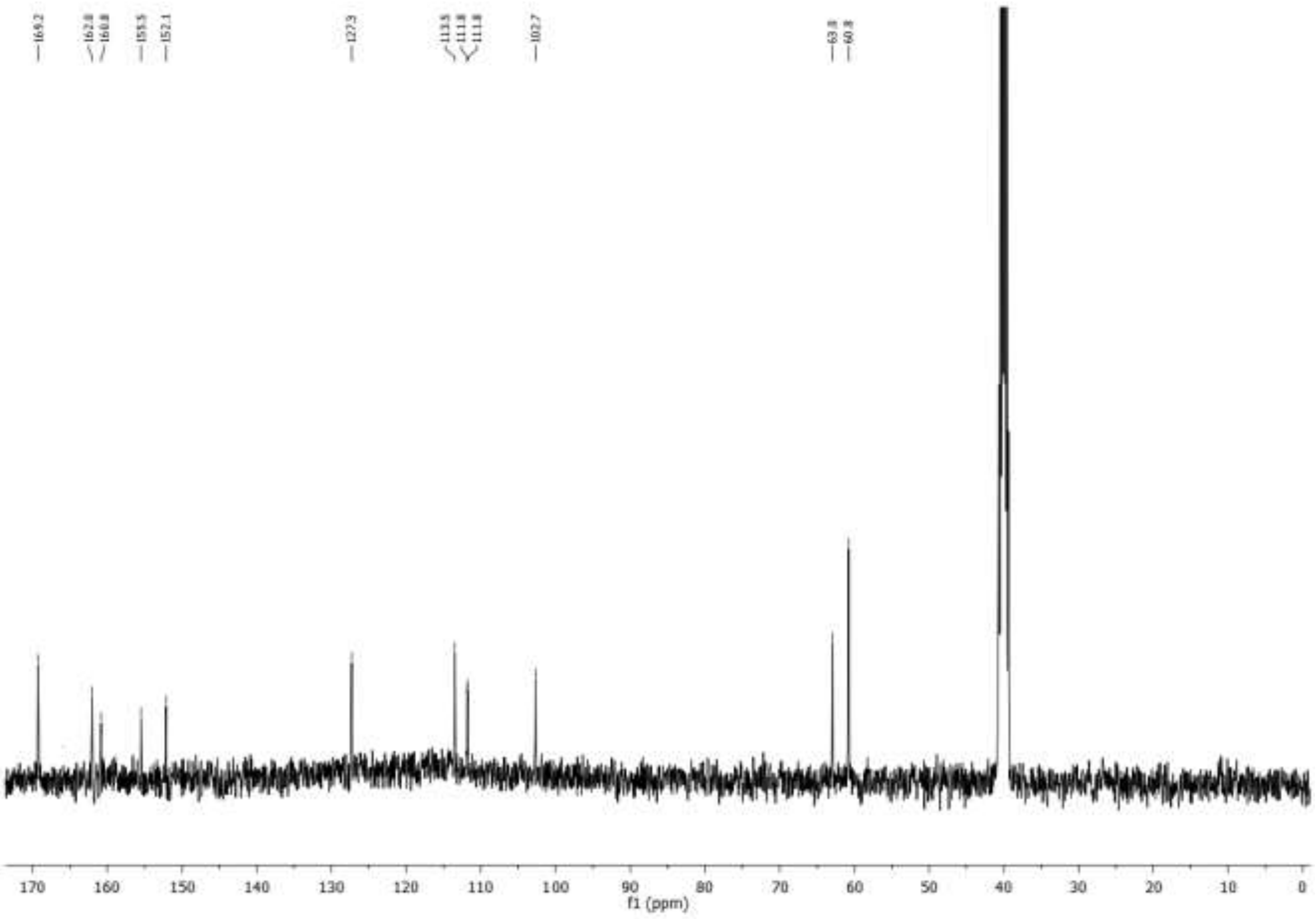

S1B Fig. Spectral properties of CT51. ^{13}C-NMR spectrum.

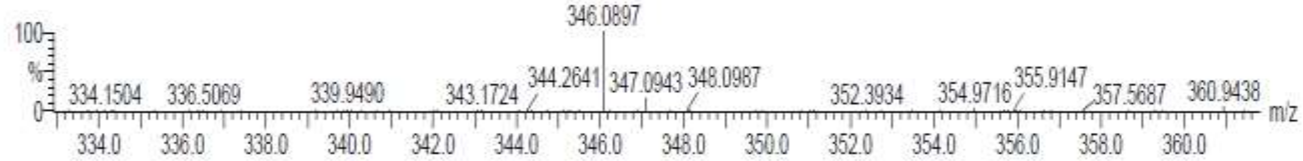

	Mass	Calc. Mass	mDa	PPM	DBE	i-FIT	i-FIT (Norm)	Formula
Minimum:					-1.5			
Maximum:			5.0	5.0	50.0			
	346.0897	346.0903	-0.6	-1.7	7.5	16.0	0.0	C15 H17 N O7 Na

S1C Fig. Spectral properties of CT51. ESI-MS analysis.

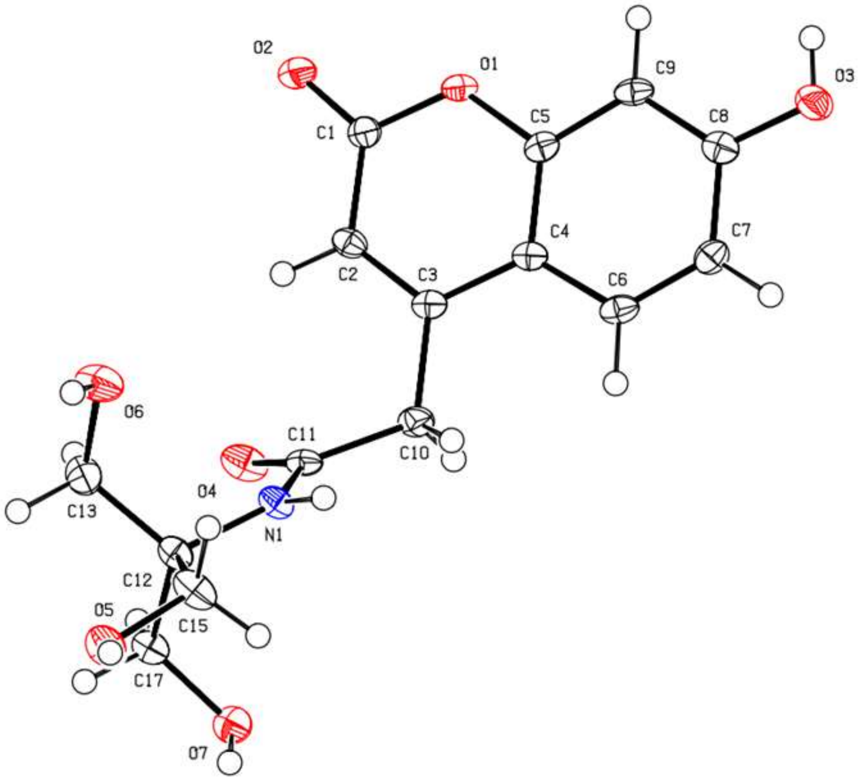

S1D Fig. Crystal structure of CT51.
