# Supplementary figures and images for: Development of an iron-selective antioxidant probe with protective effects on neuronal function
Source: PLoS One. 2017 Dec 11;12(12):e0189043. doi: 10.1371/journal.pone.0189043 (PMC5724820; doi:10.1371/journal.pone.0189043)

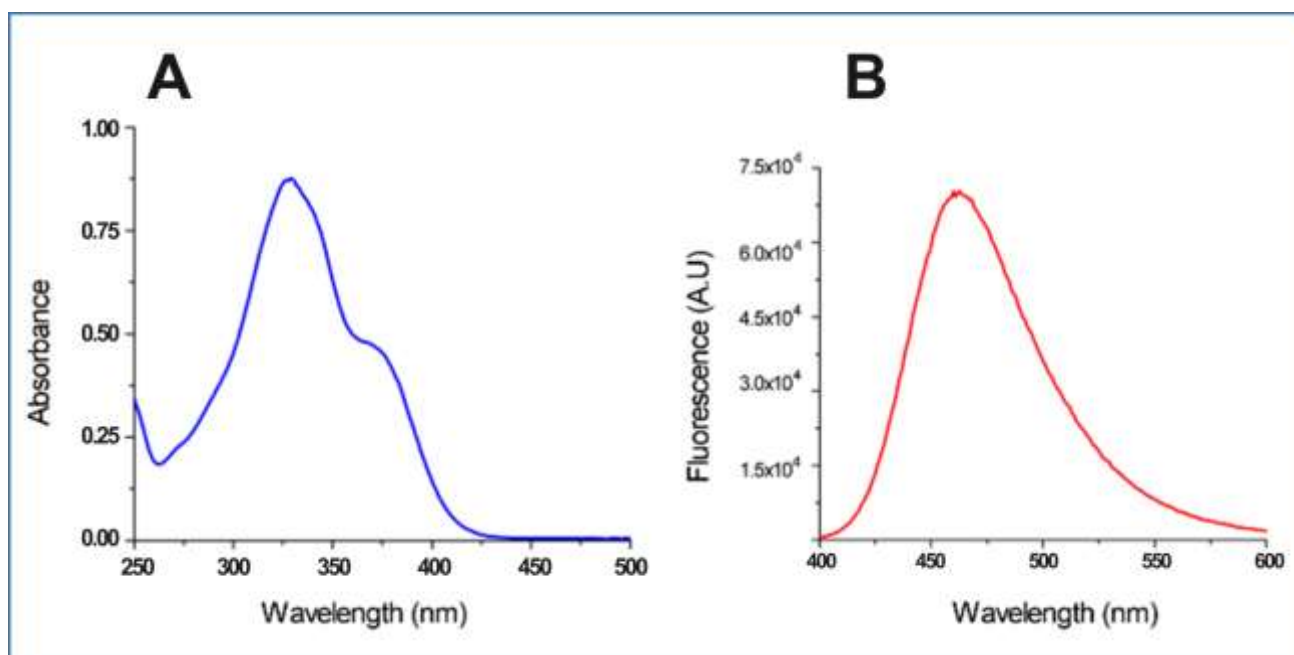

S2 Fig. (A) Absorption spectra and (B) emission spectra of CT51.

Supplement: S2 Fig — (A) Absorption spectra. (B) Emission spectra. (PDF) [file pone.0189043.s002.pdf]

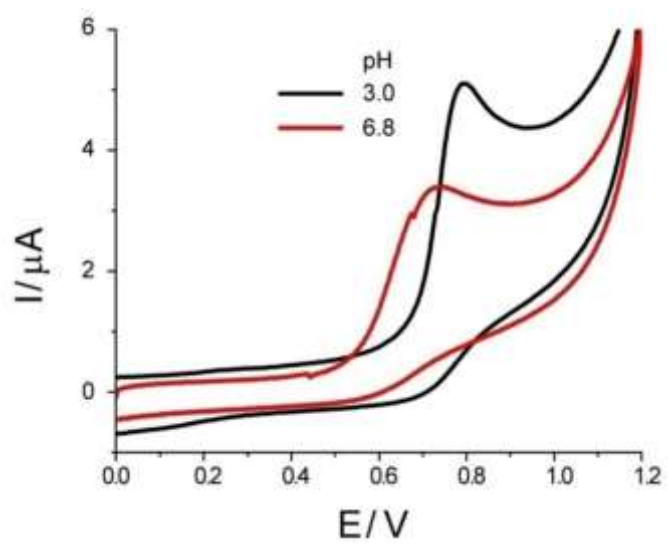

**S4 Fig. Voltammograms of CT51.**

Supplement: S4 Fig — Voltammograms of CT51 (0.96 mM) determined at pH 6.8 (red line) or pH 3.0 (black line). To perform the cyclic voltammetry analysis, 7.5 mL of ultrapure water, 0.5 mL Phosphate-buffered saline, pH 3.0 or 6.8, and 0.25 mL of 1.54 mM CT51 were added to the electrochemical cell. After a resting time of 3 s, the voltammograms were performed at a scan rate of 100 mV s-1. (PDF) [file pone.0189043.s004.pdf]
