# Supplementary material for: Development of an iron-selective antioxidant probe with protective effects on neuronal function
Source: PLoS One. 2017 Dec 11;12(12):e0189043. doi: 10.1371/journal.pone.0189043 (PMC5724820; doi:10.1371/journal.pone.0189043)
Supplement: S3 Fig — (A) Stern-Volmer relation for CT51 in the presence of Fe2+. Deactivation of fluorescence was analyzed by the Stern-Volmer equation. (B) Benesi-Hildebrand plot for CT51 with added Fe2+. Fluorescence intensity data for the CT51-Fe2+ complex were plotted according to the Benesi-Hildebrand equation. (PDF) [file pone.0189043.s003.pdf]

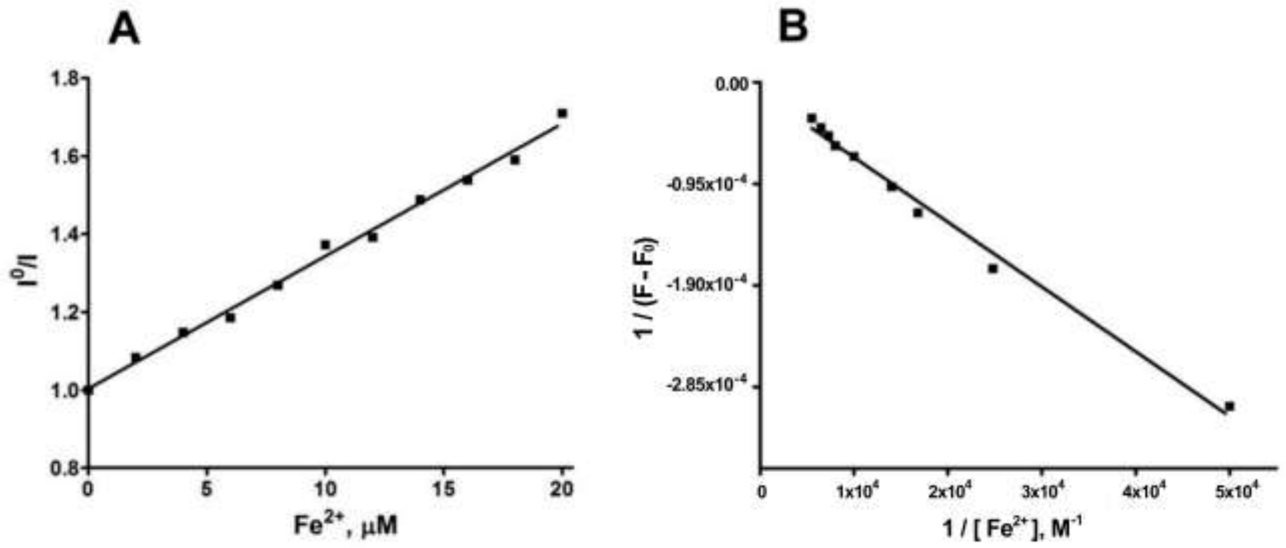

S3 Fig. (A) Stern-Volmer relation for CT51 in the presence of Fe^{2+}. (B) Benesi-Hildebrand plot for CT51 with added Fe^{2+}.
