# Supplementary material for: Development of an iron-selective antioxidant probe with protective effects on neuronal function
Source: PLoS One. 2017 Dec 11;12(12):e0189043. doi: 10.1371/journal.pone.0189043 (PMC5724820; doi:10.1371/journal.pone.0189043)
Supplement: S5 Fig — (A) Immunofluorescence of 4-HNE-protein adducts formed under basal culture conditions (Control) and in cultures treated for 24 h with the oxidative stress inductor rotenone (3 μM), in the absence or presence of 250 or 500 nM of the Cu+1 chelator bathocuproine (BC) or CT51. The figure shows images from representative experiments out of 2 independent determinations. Rotenone treatment increased cell immunostaining of 4-HNE-protein adducts. This increase was partially prevented by 250 nM CT51 and completely prevented by 500 nM CT51 but not by 250 or 500 nM bathocuproine. To appreciate these differences better, the immunostaining intensity was transformed into a thermal scale with the ImageJ program (right-hand color bar), in which fluorescence intensity increases from blue to red to white. (B) Quantification of fluorescence intensity determined in 25–45 cells per experimental condition. Values represent Mean ± SEM. Significance of differences was evaluated by one-way ANOVA followed by Tukey’s post-hoc test; ns: not significant. (PDF) [file pone.0189043.s005.pdf]

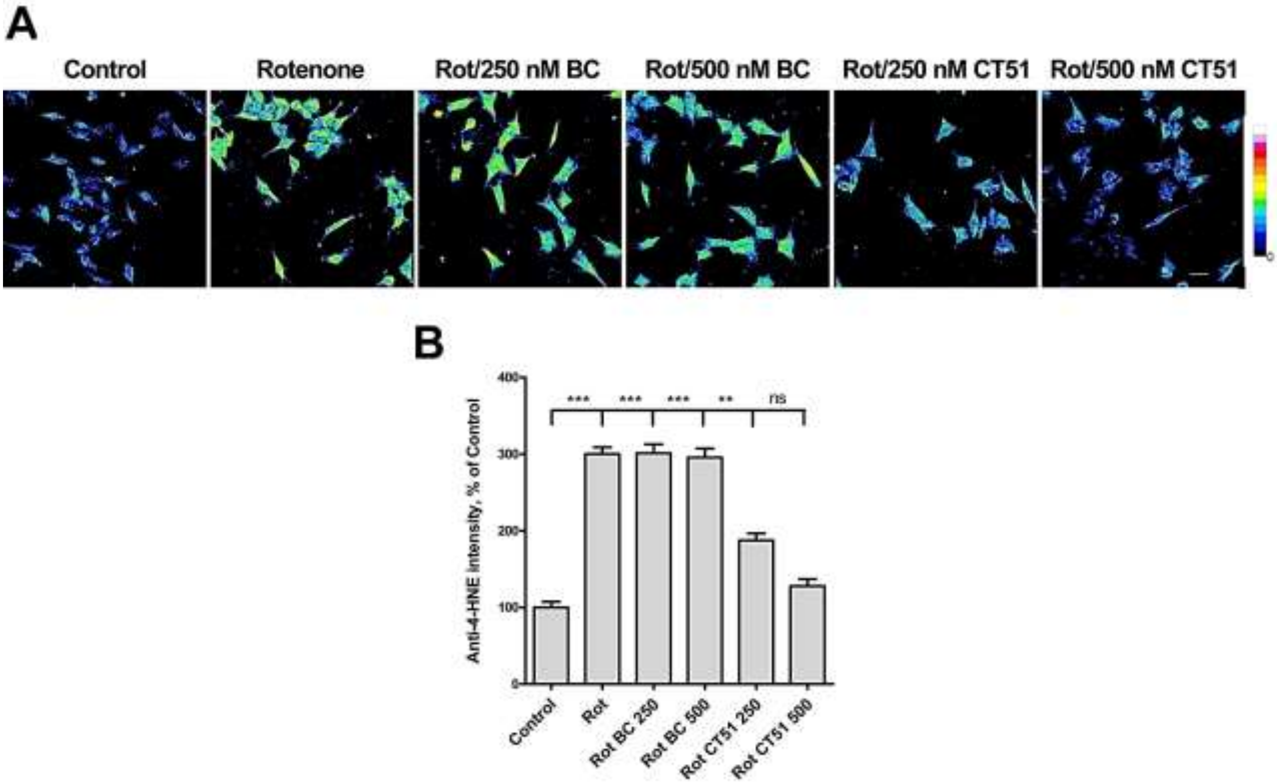

S5 Fig. Cu is not involved in rotenone-induced 4-HNE-protein adduct formation. (A) Immunofluorescence of 4-HNE-protein adducts. (B) Quantification of fluorescence intensity.
