# Supplementary material for: Development of an iron-selective antioxidant probe with protective effects on neuronal function
Source: PLoS One. 2017 Dec 11;12(12):e0189043. doi: 10.1371/journal.pone.0189043 (PMC5724820; doi:10.1371/journal.pone.0189043)
Supplement: S1 Appendix — (PDF) [file pone.0189043.s006.pdf]

S1 Appendix. Stern-Volmer equation, Benesi-Hildebrand equation and equation used to calculate fluorescence quantum yield.

(**A**) Stern-Volmer equation

$$\frac{I^0}{I} = 1 + K_{sv}\,[Q]$$

In this equation, Ksv is the Stern-Volmer constant, which is related to the accessibility of the fluorophore to the quencher (better accessibility, higher Ksv); when the extinction is collisional (dynamic), the value of this constant is equal to the product of the reaction rate constant (K_q) between the excited state and the quencher and the fluorophore lifetime (τ_0).

(**B**) Fluorescence intensity data for the CT51-Fe^{2+} complex were plotted according to the Benesi-Hildebrand equation:

$$\frac{1}{F - F_0} = \frac{1}{(F_{max} - F_0)\,[Fe^{2+}]} + \frac{1}{F_{max} - F_0}$$

In this equation, K_a is the stability constant for 1:1 complex formation; F_o is the fluorescence intensity of the sensor at 460 nm (with λ excitation 329 nm) in the absence of Fe^{+2}. F is the observed fluorescence intensity at 460 nm as a function of varying Fe^{+2} concentration and F_{max}, the maximal fluorescence intensity measured at 460 nm in the presence of Fe^{2+}. Values for the y-axis intercept and the slope were, respectively, (-1.02 ± 0.30) x 10^{-6} and (-6.76 ± 0.20) x 10^{-9} M.

(**C**) Calculation of fluorescence quantum yield.

$$\Phi_S = \Phi_r\left(\frac{A_r F_s}{A_s F_r}\right)\left(\frac{\eta_s^2}{\eta_r^2}\right)$$

The fluorescence quantum yield (Φ) was determined using quinine sulfate dissolved in 0.5 M H_2SO_4 (Φr = 0.546) as standard. The subscripts s and r in this equation denote sample and reference, respectively. A is absorbance at the excitation wavelength (very dilute solutions), F is the integrated fluorescence intensity, and η is the refractive index of the medium.
