# Supplementary material for: Development of an iron-selective antioxidant probe with protective effects on neuronal function
Source: PLoS One. 2017 Dec 11;12(12):e0189043. doi: 10.1371/journal.pone.0189043 (PMC5724820; doi:10.1371/journal.pone.0189043)
Supplement: S1 Table — (PDF) [file pone.0189043.s007.pdf]

S1 Table

Crystal data and details of the structure determination (1-4)

Crystal data

2 ($C_{15}H_{17}N O_7$), CH_4O	δ = 86.298(3) º
Mr = 678.63	V = 1518.6(2) $Å^3$
Triclinic, P-1 (Nº 2)	Z = 2
a = 7.7008(6) Å	Mo$K\alpha$ radiation
b =13.8964(11) Å	μ = 0.119 mm^{-1}
c =15.3172(13) Å	T = 100 K
α = 68.684(3) º	0.03 x 0.12 x 0.12 mm
β = 84.244(3) º	

Data collection

Bruker-Nonius diffractometer	5042 reflections with $I > 2\sigma(I)$
6881 measured reflections	R_{int} = 0.113
6881 independent reflections	Theta min-max (deg) 1.6-27.6

Refinement

R [($F^2 > 2\sigma(F^2)$] = 0.0654	H-atom parameters constrained
$wR(F^2)$ = 0.1852	444 parameters
S = 1.06	$\Delta\rho_{max}$ = 0.75 e $Å^{-3}$
F(000) = 716	$\Delta\rho_{mim}$ = -0.42 e $Å^{-3}$

---

1. SMART, SAINTPLUS V6.02, SHELXTL V6.10 and SADABS; Bruker Analytical X-ray Instruments Inc., Madison, Wisconsin, USA.
2. G. M. Sheldrick, 1997. SHELXL-97. Program for the Refinement of Crystal Structures. University of Göttingen, Germany.
3. K. Brandenburg, DIAMOND. Visual Crystal Structure Information System. Version 2.1e Crystal Impact GbR, Bonn, Germany, 1999.
4. A. L. Spek, *J. Appl. Cryst.,* 36: 7-13, 2003
